# Assessment of Pyrogenic Response of Medical Devices and Biomaterials by the Monocyte Activation Test (MAT): A Systematic Review

**DOI:** 10.3390/ijms25147844

**Published:** 2024-07-18

**Authors:** Izabela Gimenes, Janaína Spoladore, Bruno Andrade Paranhos, Tea Romasco, Natalia Di Pietro, Adriano Piattelli, Carlos Fernando Mourão, Gutemberg Gomes Alves

**Affiliations:** 1 Post-Graduation Program in Science and Biotechnology, Fluminense Federal University, Niteroi 24220-900, Brazil; izabela.gimenes@incqs.fiocruz.br (I.G.); janspoladore@gmail.com (J.S.); 2 Carlos Chagas Filho Biophysics Institute, Federal University of Rio de Janeiro, Rio de Janeiro 21941-170, Brazil; paranhosba@biof.ufrj.br; 3 Division of Dental Research Administration, Tufts University School of Dental Medicine, Boston, MA 02111, USA; tea.romasco@unich.it; 4 Department of Medical, Oral and Biotechnological Sciences, “G. d’Annunzio” University of Chieti-Pescara, 66100 Chieti, Italy; 5 Center for Advanced Studies and Technology (CAST), “G. d’Annunzio” University of Chieti-Pescara, 66100 Chieti, Italy; 6 School of Dentistry, Saint Camillus International, University of Health and Medical Sciences, 00131 Rome, Italy; apiattelli51@gmail.com; 7 Department of Periodontology, Tufts University School of Dental Medicine, Boston, MA 02111, USA; 8 Cell and Molecular Biology Department, Institute of Biology, Fluminense Federal University, Niteroi 24220-900, Brazil; gutopepe@yahoo.com.br

**Keywords:** monocyte activation test, biomaterials, biocompatibility, pyrogens, graft materials, regeneration

## Abstract

Pyrogens are fever-inducing substances routinely investigated in health products through tests such as the Rabbit Pyrogen Test (RPT), the Limulus Amebocyte Lysate (LAL), and the Monocyte Activation Test (MAT). However, the applications of the MAT for medical devices and biomaterials remain limited. This work aimed to overview the studies evaluating the pyrogenicity of medical devices and biomaterials using the MAT, highlighting its successes and potential challenges. An electronic search was performed by December 2023 in PubMed, Scopus, and Web of Science, identifying 321 records which resulted in ten selected studies. Data were extracted detailing the tested materials, MAT variants, interferences, and comparisons between methods. Methodological quality was assessed using the ToxRTool, and the results were synthesized descriptively. The selected studies investigated various materials, including polymers, metals, and natural compounds, employing the different biological matrices of the MAT. Results showed the MAT’s versatility, with successful detection of pyrogens in most materials tested, though variability in sensitivity was noted based on the material and testing conditions. Challenges remain in optimizing protocols for different material properties, such as determining the best methods for direct contact versus eluate testing and addressing the incubation conditions. In conclusion, the MAT demonstrates significant potential as a pyrogen detection method for medical devices and biomaterials. However, continued research is essential to address existing gaps, optimize protocols, and validate the test across a broader range of materials.

## 1. Introduction

Innovative healthcare products, such as biomaterials and medical devices, are continually being improved, with new products regularly entering the market [1,2,3]. In clinical and dental practice, many medical devices are either permanently inserted into the body or come into temporary contact with blood or tissues, presenting potential risks to patients. Hence, they must be free from contaminants [4].

Contamination by pyrogens is a significant concern for health surveillance as it can induce fever, alter the hemostatic response, cause shock, and even result in death [4,5,6,7]. Such contamination often results from microorganism proliferation during or after production, with bacterial endotoxin or lipopolysaccharide (LPS) from Gram-negative bacteria being the most common contaminants [4,8]. The multiple steps required in producing different materials increase the risk of endotoxin incorporation through environmental contamination or the use of non-endotoxin-free components [8]. Therefore, sterilization techniques, though essential, are insufficient to guarantee the absence of pyrogens, rendering it necessary to evaluate the presence of pyrogens during the quality control of produced batches [4,9].

Pyrogens can be classified as either endogenous (produced internally), such as the cytokines Interleukin-1 beta (IL-1β), Interleukin-6 (IL-6), and Tumor Necrosis Factor-alpha (TNF-α), or exogenous (originating outside the body), including viruses, yeasts, and fungi, as well as environmental particles collected from outdoor and indoor air (between PM10-2.5 and PM2.5 in outdoor and indoor air) and bioaerosols that may carry pyrogenic contaminants [10,11,12,13]. Material-mediated pyrogens (MMPs) represent another relevant class of non-endotoxin exogenous pyrogens, which consist of any non-biological substance released from medical devices that may initiate a pyrogenic response. Examples of MMPs include residues from manufacturing processes, such as cutting fluids, mold releases, cleaning agents, and processing aids. Examples of MMPs implicated in pyrogenicity are various metals like nickel salts and fine metal particulates, including titanium, titanium alloy, and stainless steel [14,15].

Currently, three tests are recommended for evaluating pyrogenicity: the Rabbit Pyrogen Test (RPT), the Bacterial Endotoxin Test (BET, also known as the Limulus Amebocyte Lysate test or LAL), and the Monocyte Activation Test (MAT) [15]. The RPT was the first regulatory method for evaluating injectable products, introduced in the 1940s, and is accepted in several Pharmacopoeias [8,16,17,18,19]. This method identifies the presence of both endotoxin and non-endotoxin pyrogens by the fever response [20]. The product is injected into a rabbit’s ear vein, and the temperature variation is monitored via a rectal thermocouple [21]. However, testing solid products such as medical devices using the RPT presents complications. The insertion of the material into the animal’s skin can lead to tissue damage, inflammation, and fever that arise from the wound unrelated to the contamination itself. This method, therefore, risks producing erroneous results. The tissue destruction during implantation into the animal may itself cause an inflammatory response that does not necessarily reflect to pyrogenic contamination of the material.

Additionally, performing the RPT with eluates resulting from washing the test product is considered to underestimate the magnitude of contamination [15,22]. The extraction efficacy is influenced by variables such as temperature, shaking intensity, and storage conditions, raising concerns about the solubilization efficiency of pyrogens in this procedure. Furthermore, it is not well understood whether pyrogens that adhere to surfaces and those in solution exhibit different inflammatory properties [22]. To mitigate these issues, alternative approaches can be adopted, such as optimizing the extraction process, utilizing more sensitive analytical techniques, or directly testing the material itself rather than relying solely on eluates to achieve a more accurate evaluation of contamination levels [22].

Therefore, international standards for systemic toxicity of medical devices, such as ISO 10993-11:2017 [23], do not recommend using the RPT for new products due to ethical and technical considerations and the push towards reducing animal testing, even though chemical entities with unknown pyrogenic potential still need to be evaluated for MMPs [15]. Recently, the European Pharmacopoeia banned the RPT from its monograph, reflecting the growing trend of avoiding animal use in pyrogenicity testing. Despite the need to assess chemicals with unknown pyrogenic potential, alternative methods are being favored to align with the 3R principles (Replacement, Reduction, and Refinement), promoting more humane and scientifically advanced testing approaches.

The BET, also known as the LAL test, is an in vitro method introduced in the 1980s that detects endotoxins through the detectable gelling reaction of the horseshoe crab hemolymph when in contact with endotoxins from Gram-negative bacteria. However, the application of the BET is restricted to aqueous samples, requiring solid materials to be tested via solid eluates [24]. Despite its usefulness, the BET possesses several limitations in specificity to endotoxins from Gram-negative bacteria which may overlook pyrogens of other origins. Additionally, the BET’s extraction efficiency may vary, affecting the accuracy and consistency of the results. The factors collectively make it less suitable for comprehensive assessment of pyrogenicity in biomaterials and medical devices, highlighting the need for alternative or supplementary testing methods [15].

An alternative to the RPT and the LAL, the MAT, initially developed in the 1990s as a Non-Animal Methodology (NAM), was lately introduced in the European Pharmacopoeia and recognized by several regulatory bodies around the world [5,14,25,26]. This method detects or quantifies substances that activate human monocytes, monocytic cell lines (MONOMAC-6 or MM6), or Peripheral Blood Mononuclear Cells (PBMCs) by the release of endogenous mediators (TNF-α, IL-1β, and IL-6) known for the initiation of the pyrogenic response [5,26,27,28,29,30]. This test has been validated for injectable health products, while its application to other domains must be evaluated on a case-by-case basis, considering each material’s intrinsic characteristics and potential for interference [6], including their chemical composition, surface properties, size, degradation products, manufacturing residues, sterilization methods, and interactions with biological matrices. Regarding biomaterials and medical devices, while their properties differ significantly from those of injectable products, existing studies have demonstrated the potential for assessing biomaterial’s pyrogenicity using MAT [15].

In this context, the present review aims to provide a comprehensive overview of studies evaluating the pyrogenicity of medical devices and biomaterials using the MAT, highlighting the successes and potential methodological challenges to contribute to best practices in pyrogen testing and safety assessment of medical devices.

## 2. Methods

### 2.1. Protocol and Registration

This systematic review was conducted following the guidelines of the Preferred Reporting Items for Systematic Reviews and Meta-Analyses (PRISMA), which consist of a list of 27 items that guided this review (Supplementary Table S1). The study protocol was registered in the Open Science Framework database, accessible via the following link: osf.io/qunwf (accessed on 6 June 2024).

### 2.2. Research Question and Eligibility Criteria

The qualitative research question was formulated using a PIO strategy, where Population (P) = medical device OR biomaterial; Intervention (I) = application of the Monocyte Activation Test; and Outcome (O) = detection of pyrogenicity. The eligibility criteria for selecting studies were based on this framework. Inclusion criteria encompassed in vitro studies conducted on biomaterials, dental medical materials, and medical devices using the MAT or one of its variants, with a detailed exposure methodology. Exclusion criteria encompassed studies that reported data not involving biomaterials or medical devices (wrong population), studies applying only pyrogen tests such as RPT, LAL, Factor C (rFC), or BET other than MAT (wrong study design), and publications types like case reports, reviews, observational studies, letters to editors, editorials, commentaries, conference abstracts, and book chapters that did not present primary data (wrong publication type).

### 2.3. Search Strategy

An electronic search was conducted up to December 2023 in the Databases PubMed (NCBI) (http://www.ncbi.nlm.nih.gov/sites/pubmed) (accessed on 15 May 2023), Scopus (Elsevier) (http://www.scopus.com) (accessed on 25 May 2023), and Web of Science (Clarivate) (https://www.webofknowledge.com) (accessed on 1 June 2023), using the search strategy presented in Table 1. A manual search of the reference lists of articles selected for the systematic review was also performed to detect relevant publications missing from the research database. There was no need to contact the authors for missing documents.

### 2.4. Study Selection

The retrieved documents were exported and organized into the RayyanTM Web and Mobile App for Systematic Reviews (2016, Cambridge, MA, USA) reference management software (https://www.rayyan.ai, accessed on 3 June 2023), where duplicates were manually removed. Three authors independently evaluated the titles and abstracts (J.S., I.G., B.A.P.) to determine whether the articles met the inclusion and exclusion criteria according to the PIO strategy. Selected articles were read in full to confirm eligibility. In case of doubts or disagreements regarding the inclusion/exclusion of an article among the reviewers, a consensus meeting with a third reviewer (G.G.A.) was held. The reasons for excluding articles were recorded after analyzing the full text.

### 2.5. Data Collection

Data from all studies were extracted by three authors (J.S., I.G., B.A.P.) and organized into Microsoft Excel (Excel 2010^®^, Microsoft^®^, Redmond, WA, USA) for further analysis. The extracted information included the following: author, year of publication, type of material tested, intervention performed, incubation time or pyrogen extraction method, form of contamination, and the variant of the MAT used. For studies involving fresh or cryopreserved blood, details such as the amount of blood used and the number of donors were recorded. Additionally, the data encompassed the specific cytokine analyzed, comparisons with other tests, cell viability, protocol modifications, and outcomes. These data were used to conduct a descriptive analysis, which provided the foundation for the qualitative discussion of the main findings.

### 2.6. Study Risk of Bias Assessment

Two independent reviewers (I.G.L., B.A.P.) assessed the methodological quality of the included studies in compliance with the Toxicological Data Reliability Assessment Tool (ToxRTool) criteria [31]. In cases of doubt, a third reviewer (G.G.A.) would mediate to solve conflicts and inconsistencies. The ToxRTool for in vitro studies consists of an 18-point rating checklist, considering methodological aspects of each study, such as identification of the test substance and test system, study design, and result documentation. Articles with less than 11 points are considered unreliable, studies with 11–14 points are reliable with possible restrictions, and studies with 15–18 points are considered reliable without restrictions.

### 2.7. Synthesis of Results

The characteristics of the included studies were summarized and tabulated using Excel spreadsheets (Excel 2010^®^, Microsoft, Redmond, WA, USA). The studies were grouped for the synthesis based on the outcomes of interest related to the qualitative parameters of pyrogenic response. Subsequently, the characteristics of the studies were screened to determine which were similar enough to be grouped within each comparison, exploring and comparing the PIO elements across the studies. Data were analyzed and interpreted qualitatively to integrate the reported information and present the synthesis results descriptively.

## 3. Results and Discussion

### 3.1. Study Selection

**Figure 1:**
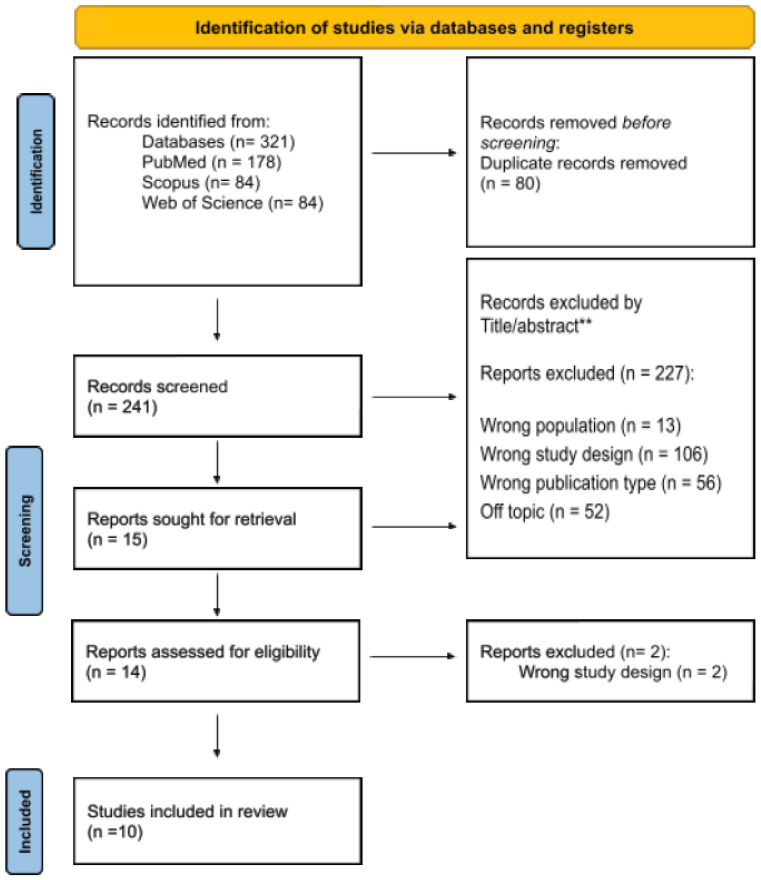
Flow diagram of the screening and selection process, according to the Preferred Reporting Items for Systematic Reviews and Meta-Analyses (PRISMA) statement. ** Excluded by Title/Abstract.

The initial search identified 321 records, as shown in Figure 1. After removing duplicates, a total of 241 records were evaluated. Based on the eligibility criteria, 226 records were excluded for the following reasons: wrong population (n = 13), wrong study design (n = 106), wrong publication type (n = 56), and off-topic (n = 52). A total of 14 studies were selected for a full reading. One publication, not initially identified in the search key, was included in the list of studies [32]. Out of the 14 eligible studies, two were excluded due to the wrong study design, and three were excluded as off-topic. Therefore, ten studies were ultimately included in this review.

### 3.2. Study Characteristics

Information was extracted from the ten selected works, and their main characteristics are summarized in Table 2. Most of the studies (seven) compared the MAT with the LAL [4,22,32,33,34,35,36]; four studies compared with the RPT [33,34,36,37]. Regarding the test matrix, nine studies evaluated the products using fresh blood [4,22,32,34,35,36,37,38,39], while only two studies evaluated cryopreserved and fresh blood [35,36], and one study assessed fresh blood and PBMCs [39]. In addition, one study used MM6 cell culture in place of blood [33], and another employed the subclone MM6-CA8 [34]. IL-1β was the main cytokine detected for pyrogenicity, as a single study did not evaluate this interleukin [34].

All studies used standard endotoxin stimuli as a positive control, including LPS from *E. coli* O-113 [4,22,35,39], *E. coli* O111:B4 [32,35], *E. coli* O55:B5 [33,34,37], and *E. coli* UKT-B [33]. Two authors did not inform the strain of LPS/endotoxin used [36,38]. Non-endotoxin pyrogen stimuli were also assessed in some studies, including Zymosan [22,35,39], Peptidoglycan (PGN) [33,35], and Lipoteichoic acid (LTA) from different strains, like *S. aureus* (SaLTA) [4,35], *B. subtilis* (BsLTA) [35,37], and *S. aureus* (pSaLTA) [35]. One study also included plant-derived Phytohaemagglutinin lectin from *Phaseolus vulgaris* (PHA) and chemical pyrogen 2,4,6-trinitrophenol [37]. Another study employed culture suspensions of *P. putida*, *S. epidermidis*, and *E. coli* [32].

Among the ten studies reviewed, seven utilized extract preparation as their exposure protocol, while six employed direct contact methods, with some studies incorporating both approaches. Notably, at least two of these studies developed novel devices or systems specifically designed to enhance the evaluation of pyrogenicity in biomaterials [4,35].

### 3.3. Risk of Bias in Studies

The methodological quality assessment of the included studies is reported in Table 3. Five studies were considered “reliable without restriction” [4,22,32,34,39] with good methodological quality, and five were reliable with restriction [33,35,36,37,38] according to ToxRTool criteria [31]. The main report limitation was related to poor or no identification of the tested materials, including sources, composition, and batches.

### 3.4. Synthesis of Results

A narrative synthesis was performed and is presented in the following discussion, with results grouped according to the type of parameter evaluated by the studies, which involve the category of analyzed materials, the types of exposure protocols, the choice for biological matrix, the type of pro-inflammatory cytokines assessed for pyrogenicity, contamination, and interference assessments, and the correlation of results with LAL or RPT.

### 3.5. Discussion

Medicine has advanced significantly, embracing new frontiers in therapies and the development of cutting-edge equipment, medical tools, and materials. This progress demands meticulous safety evaluation to ensure that these innovative tools are harmless and compatible with the human body. Critical to this assessment is the need to ensure that products are free from contamination by pyrogens and harmful materials or fluids encountered during manufacturing processes. For instance, heat sterilization methods such as autoclaving or dry heat sterilization may occasionally fail to completely eliminate pyrogenic substances from equipment or materials used in production or packaging, which may contain residual chemicals or contaminants capable of acting as pyrogens upon contact with parenteral drugs or medical devices [40]. Given these challenges, it is essential that mandatory safety tests, like the Pyrogen Test, extend their scope to encompass not only traditional injectable products but also a wide array of solid materials, including medical devices such as implants, medical plastics, and dialysis machines [14].

The MAT is a NAM that employs human biological components, including whole blood (fresh or cryopreserved), blood fractions like PBMCs, or MM6, and its clones like MM6-CA8 to test for pyrogenicity [41]. Although theoretically, the MAT can test any material regardless of its shape and size, regulatory bodies currently recommend conducting a validation study to determine its suitability for a specific substance or material [42,43]. Once the MAT has been adapted and validated for a particular applicability domain, it should be consistently applied throughout the various stages of production and for the final product. This ongoing application is crucial as it ensures that materials are monitored for pyrogenic contaminants that could be introduced during manufacturing processes. Despite the availability of widespread sterilization methods, the MAT remains a critical tool for confirming the effectiveness of these processes and identifying whether more efficient alternatives can be implemented for medical devices.

The MAT was officially recognized by the Food and Drug Administration (FDA) for testing parenteral drugs in 2009 and by the European Pharmacopoeia in 2010, endorsing it as a substitute for rabbit testing, provided specific product validation was conducted [14]. The test was accepted as a replacement for the LAL test in medical devices by 2012, and ISO 10993-1:2018 [44] expressed a preference for MAT, depending once again on product-specific validation. Nevertheless, the ISO still recommends further studies to validate the detection of MMPs that MMPs can induce pyrogenic responses through mechanisms independent of the Toll-like receptors (TLRs) signaling pathway [14]. Furthermore, the Organization for Economic Cooperation and Development (OECD) has published Guide 34 to provide guidance on issues related to the validation of new or updated test methods, including the MAT. However, for new materials, a product-by-product validation is recommended, which functions as a batch validation. These requirements impact the practical use of the MAT by ensuring that the test is scientifically valid, reliable, and relevant for assessing the pyrogenic potential of materials in medical products. Compliance with these requirements also enhances the international acceptance of MAT as an effective alternative method for safety and quality testing, reducing the need for animal testing and improving the safety of medical products for patients. Therefore, understanding the adequacy of the MAT for a great diversity of materials and devices, their exposure methods, and the results obtained is essential for its future application in industry.

#### 3.5.1. Device Categories

Medical devices interact with human tissues or the bloodstream in various ways, often coming into temporary contact or being implanted through surgical procedures. Depending on their intended use, these implants can be designed for permanent placement, scheduled for later removal, or engineered to be partially or completely absorbed by the body over time [45]. The functionality and suitability of these devices are influenced by their composition, surface properties, and structural design [35]. Therefore, comprehensive testing, including pyrogen testing with methods like the MAT, is essential to evaluate these characteristics and ensure the safety and efficacy of medical devices across their diverse applications.

Not all materials are listed for pyrogenicity testing under the FDA. In this sense, adequacy tests need to be carried out with a special focus on devices that are already routinely used in health centers and for those materials that are immunogenic (such as those containing collagen/chitosan, polyethylene glycol, or xenografts), and for which expanding the applicability of tools such as the MAT becomes a high priority. Most studies in this review (70%) investigated implantable materials of non-metallic origin. One study evaluated intraocular lenses made of different materials, such as silicone, hydrophobic and hydrophilic acrylic, and methyl methacrylate [32]. Three studies evaluated polymeric materials [4,36,37]. Three studies evaluated biomaterials of natural compounds, such as latex and silicone [33], collagen, alginate, chitin, and poly-L-leucine [34], and cotton-based surgical invasive devices [39]. Four studies included implantable medical devices of metallic origin [4,22,35,38], such as cobalt-chromium compounds [4], stainless steel [4,35], steel [35], titanium alloy (Ti6Al4V) [22,35], titanium [35,38], and zirconia [38]. Interestingly, despite the significant role of ceramic materials as grafting agents in various biomedical applications, the adequacy of MAT for testing pyrogenicity on this class of material was not assessed in those studies. This is particularly important given the growing use of ceramics in regenerative medicine and their potential for prolonged contact with biological tissues. Their absence in the reviewed studies highlights a gap in the current research, underscoring the need for future investigations into the pyrogenicity of ceramic biomaterials using MAT.

#### 3.5.2. Exposure Protocols

Different types of devices may require optimization in executing the methodology according to the material’s nature, size, and surface, either using extracts (eluate) or by direct contact with the test matrix. Brown et al. [15] reported a discussion on this theme at a workshop at the National Institutes of Health (NIH) in 2018, including representatives from MAT testing laboratories, medical device manufacturers, FDA, United States Pharmacopeia (USP), ISO, and experts in the development of MAT protocols. The report indicated a consensus on the division of materials by size, where small samples would fit in a tube of up to 10 mL, allowing the material to be immersed in complete contact with the test matrix. When direct contact is performed, the authors emphasize that the protocol should specify additional procedures, such as the use of static (without any agitation or reactors) or dynamic incubation (with continuous or periodic agitation), and account for the incubation period, which may alter the sensitivity of test detection. The debate also led to the recommendation of the development of specific reference standards for devices, as advocated by reference institutions such as USP and the National Institute of Standards and Technology (NIST) [15].

Among the selected studies in this review, some of the chronologically older studies evaluated medical devices preceded the validation studies and regulatory acceptance of the original MAT method. Haishima et al. [33] and Nakagawa et al. [34] investigated, between 2001–2003, an activation test using the MM6-CA8 cell line; however, this was conducted with a limited description of the exposure methods. Therefore, the studies only demonstrated the ability of the method to be sensitive to the test system. Mazzotti et al. [22] conducted biological assays using MAT through the traditional protocol, involving the direct contact of fresh and cryopreserved blood with neurosurgical implants. The study was designed to determine if the in vitro system is sensitive to various types of contamination, including LPS, Zymosan, and manual contamination by workers without gloves. In this design, metallic clips were deliberately contaminated with 5 pg of LPS in both cryopreserved and fresh blood. The results showed that both matrices had the same sensitivity and a recovery rate within 50%, indicating that the clip does not interfere with the test system. The authors concluded that titanium aneurysm clips are a safe solid biomaterial that does not interfere with the MAT test system. It is interesting to notice that this protocol from 2007 is equivalent to that lately proposed for small devices, according to Brown et al. [15], as the tests were carried out with the clips immersed in 5 mL tubes [22].

Werner et al. [32] analyzed extracts of different brands of intraocular lenses (IOL) in fresh blood by monitoring the release of IL-1β. A difference in this work was the use of bacterial suspension cultures (CFU/mL), Gram-negative (*E. coli* and *P. putida*), and Gram-positive (*S. epidermidis*) as contaminants of the materials. The authors obtained conflicting results for the LAL and the MAT using the same eluate in both tests, as the LAL did not detect endotoxins in the IOL-adsorbed samples. The MAT, on the other hand, presented a positive result with a dose-dependent response, being considered more sensitive and considered by the authors as more suitable for this kind of device [32].

Trunk et al. [39] evaluated cotton swabs used in surgeries by performing the traditional MAT protocol, utilizing both fresh blood and PBMCs, and assessed the levels of IL-1β and IL-6 under various conditions. The study included contamination through direct contact with a small volume (10 µL) of LPS or Zymosan solutions, followed by different sterilization treatments. The results from the whole blood tests indicated an inflammatory response, with increased release of IL-1β and IL-6 directly related to the cotton-based materials. Notably, the different sterilization treatments did not reduce the degree of activation, which contrasted with existing literature on the efficiency of sterilization processes. In contrast, the PBMC-based tests revealed differences in inflammatory activity after various sterilization processes. However, there was no reproducible effect on samples intentionally contaminated with known concentrations of LPS for IL-1β. The results demonstrated greater reproducibility in the studies using PBMCs and IL-6, where the inflammatory response was significantly reduced in 95.1% of the untreated non-sterile swabs, deeming them safe for use. Similar observations were made for materials contaminated with LPS. Additionally, the study proposed sterilization protocols to at least mitigate the inflammatory reaction induced by leukocytes, although it highlighted that the crosstalk between cells and plasma proteins in the inflammatory cascade activation in response to these materials could not be entirely isolated [39].

Other studies identified in this review proposed changes to the original MAT protocol to achieve better results for medical devices. Banerjee and Mohanan [37] and Mohanan et al. [36] analyzed eluates of polymeric gelatinous materials, proposing modifications such as reducing the incubation time to three hours and using a direct contact method between the test materials and fresh human whole blood, which enhances the detection of both endotoxin and non-endotoxin pyrogens. The study demonstrated that the MAT, particularly with a sensitive IL-1β enzyme-linked immunosorbent assay (ELISA), could detect pyrogens with a lower limit of 10 pg/mL, offering higher sensitivity than the RPT and LAL test [36]. The authors identified that the detection system loses sensitivity for the LPS after 8 h, but this limitation did not affect the polymer samples, for which the authors reduced incubation to 3 h while retaining sensitivity, distancing itself even further from international protocols, which proposes an overnight incubation [37]. This result indicates that some materials may require less incubation time than others.

**Figure 2:**
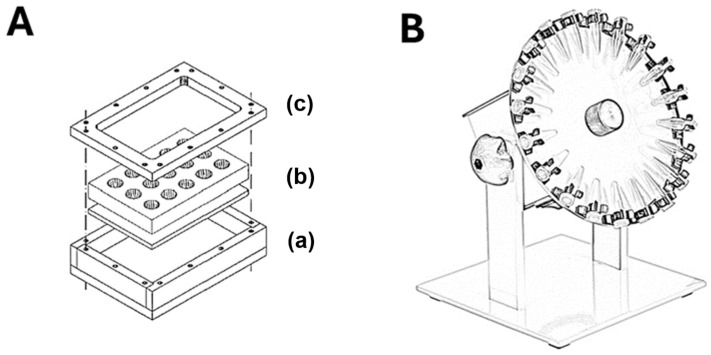
The use of devices for improved pyrogenic testing of biomaterials with MAT. (**A**) The V4A steel incubation chamber, proposed by Hasiwa et al. [35], consists of three parts, where the bottom is a tray with 14 tap holes for immersing the test material (**a**). The centerpiece, which fits onto the tray, has 15 drilled holes and is sealed with Viton-o-rings to prevent exchange between mini-chambers, allowing for both positive and negative controls to be inserted on wells of the same plate (**b**). The top part is a rectangular ring with 14 tap holes for securing with screws (**c**). (**B**) A commercially available tube rotator, similar to the one employed by Harder et al. [38] and Stang et al. [4] to generate a dynamic exposure using a tube rotator of small samples immersed in the biological matrix in test tubes, generating increased sensitivity and higher interleukin release, as reported by authors.

Some studies proposed the use of specific devices for the improvement of the MAT for biomaterials. Hasiwa et al. [35] developed a stainless steel 15-well system for performing the MAT on medical devices and biomaterials, particularly solid sheets (Figure 2A). It allows direct contact of human whole blood with the test material and supports both fresh and cryopreserved blood, making it practical for standardized testing. This design enhanced pyrogen recovery rates, especially under dynamic conditions, and was able to detect both endotoxin and non-endotoxin pyrogens, with a sensitivity up to 10 times greater at lower concentrations than compared to the eluate [35].

Harder et al. [38] and Stang et al. [4] proposed modifications to the MAT protocol, combining the use of dynamic incubation systems, as depicted in Figure 2B. Harder et al. [38] evaluated dental implants made of titanium and zirconia using gene expression analysis and an MAT performed with fresh blood, assessing IL-1β. Samples were incubated at times ranging from 1 to 24 h and kept on a rotating platform for 50 rpm for dynamic exposure using a tube rotator. Gene expression levels of TLR9, nuclear factor kappa-light-chain-enhancer of activated B cells (NFκ-B), IL-1β, and TNF-α increased after stimulation with LPS and incubation with titanium and zirconia implants. The MAT assessment showed a linear correlation between the LPS spike concentration (amount of LPS intentionally added to a sample) and IL-1β production after 8 h of incubation with fresh blood, while the method could not detect the presence of IL-1β [38]. These findings contrast with the results by Monhanan [36], which were identified at 3 h of incubation. This difference may be both a limitation due to the use of a less sensitive in-house ELISA kit by Harder et al. [38] or due to differences in the minimum incubation time for different biomaterials depending on their physicochemical nature.

Stang et al. [4] proposed two modifications in the MAT protocol when testing different biomaterials (steel plates, cobalt-chromium stents, and expanded polytetrafluoroethylene, ePTFE, vascular grafts). The first is the pyrogenic contamination (spiking) with the LPS and LTA in static liquid incubation, overnight or for up to 10 h, followed by drying at room temperature before testing. Both the LPS and LTA-spiked stents induced a low response when compared to the standard curves of the LPS and LTA. The second modification was the direct contact of contaminants on the surface of the materials by dripping and drying. This time, the MAT was effective in detecting the LPS and LTA on the dry surface of contaminated stents, while the steel plates and ePTFE grafts induced a low IL-1β signal. However, when direct contact on the drying surface was tested for the modified MAT protocol, with samples incubated under dynamic stimulation at 10 rpm with 1.2 mL of blood (1:12) overnight, all materials induced a higher interleukin release rate for the modified MAT in LPS and LTA, as compared to the standard MAT protocol [4].

#### 3.5.3. Dependance on the Biological Matrix

Monograph 2.6.30 of the European Pharmacopoeia for MAT recommends using different cell sources, like (i) whole blood obtained from a single donor or pooled whole blood, (ii) PBMC isolated from single or from pooled whole blood, and (iii) continuous human monocytic cell lines. Furthermore, the cell source intended for use in a MAT may be cryo-preserved [41].

In this review, when investigating the different biological matrices employed in the applications of MAT for material and device testing, we can see similar issues to those already discussed in the test validation for products such as injectables (vaccines, serums, and blood products). In most selected studies, the preferred matrix was fresh blood [4,22,32,34,35,36,37,38,39], which suffers from disadvantages such as (i) the small difference between test-responsive donors; (ii) the necessity of screening for pathogens in donor blood; (iii) a short 4 h window for using the freshly drawn blood, if not cryopreserved [46]. The use of PBMC also shares disadvantages regarding pathogens and short windows of use. Nevertheless, the use of fresh blood is practical, as only a small amount (100 µL) is required, and the method has fewer preparation artifacts than PBMC-based MAT [46]. It also facilitates direct contact with solid materials after adsorption to remove interference or increase sensitivity, as well as for testing medical devices on surfaces. Furthermore, different donors or pooled blood can be used, and genetic stability is not an issue, in contrast to cell line-based MAT [46].

Two studies compared the use of fresh or cryopreserved blood in MAT for solid titanium implants [22,35]. Mazzotti et al. [22] incubated aneurysm clips directly in contact with human blood. Although there was a more significant release of IL-1β in contact with cryopreserved blood, the study showed the same sensitivity in both conditions since the LPS recovery was similar. For Hasiwa et al. [35], cryopreserved blood was considered advantageous for its practicality, as it can be aliquoted and stored for long periods. Cryopreserving blood also allows for convenient running of tests before matrix utilization. However, the authors also found that cryopreserved blood was less sensitive than fresh blood. The results showed a greater release of IL-1β for cryopreserved blood when testing implant steel, titanium, and TiAl6V4, indicating that the physical nature of the material may be relevant to the nature of the chosen biological matrix.

Two studies employed cell lines as the biological matrix for the MAT. The MM6 cell line is the only validated cell line for the MAT [40], with the European Pharmacopoeia recommending its clones, like MM6-CA8, for test optimization [41]. Despite the challenges in standard cell culture practices, MM6 offers a quick, sensitive alternative to human blood tests, adhering to Pharmacopoeia guidelines [28]. While primary studies on biomaterials are lacking, most MM6 research focuses on injectable products, showing their ability to detect IL-6 release in batches that cause fever in humans [47] in a more sensitive and cost-effective way than LAL rabbit tests [48]. Among the studies selected in this review, Haishima et al. [33] used the MM6 cell line to evaluate surgical gloves and catheters, observing a good dose-response correlation of cytokine release. The authors pointed out that only peptidoglycan-positive controls elicited a weak cytokine release. The second study carried out by Nakagawa et al. [34] tested the pyrogenicity of various wound dressings made from natural biomaterials, both with fresh blood and the MM6-CA8 clone. One of the alginate extracts showed a low release of IL-6 when compared to the LAL and RPT. To understand this, the authors also evaluated human and rabbit blood for TNF-alpha. As shown by Nakagawa et al. [34], some endotoxins may be pyrogenic only for humans, being undetected by the RPT, and the cell line-based MAT may be suitable to detect them.

In this review, only Trunk et al. [39] evaluated the use of PBMCs isolated from healthy donors via venipuncture and gradient centrifugation or whole blood compared to fresh blood by testing cotton swabs for medical uses. The results pointed to greater reproducibility in tests carried out with PBMCs and IL-6 evaluation.

#### 3.5.4. Pyrogenicity Detection Parameters

The selection of cytokines for the MAT is crucial for accurately detecting pyrogens in medical devices and biomaterials. Commonly measured cytokines include IL-1β, IL-6, and TNF-α, all of which are key pro-inflammatory markers released by monocytes upon exposure to pyrogens. IL-1β is widely used due to its robust response to pyrogenic substances, making it a primary indicator in MAT assays. IL-6 and TNF-α are also frequently measured, providing a comprehensive profile of the inflammatory response. The choice of cytokine depends on the specific requirements of the test, including sensitivity and the type of pyrogens being targeted, ensuring a versatile and reliable approach to pyrogen detection.

In this review, IL-1β was the parameter of proinflammatory cytokines analyzed in 90% of the selected studies [4,22,32,33,35,36,37,38,39]. This is the best-studied cytokine that initiates the immune response in humans when an external agent penetrates the skin barrier. Haishima et al. [33] and Nakagawa et al. [34] also analyzed IL-6 and TNF-α. For both, the release was in accordance with the release of IL-1β, indicating that the application of MAT in medical devices does not cause alterations in the performance of the parameters already proposed.

It is important to note that IL-1β, IL-6, and TNF-α are not only indicators of pyrogenicity but also play significant roles in tissue regeneration, influencing processes such as inflammation, cellular recruitment, and wound healing. Therefore, their release in response to implants and bone substitutes transcends mere detection of pyrogens, providing insights into the biomaterials’ biocompatibility and potential to promote tissue repair and regeneration. This dual role underscores the importance of selecting appropriate cytokines for the MAT, as they offer a comprehensive profile of both inflammatory and regenerative responses to biomaterials, even without pyrogenic contamination.

#### 3.5.5. Endotoxin Stimuli and Interference Test

According to the Interagency Coordinating Committee on the Validation of Alternative Methods (ICCVAM), interference testing is performed to verify that a test substance does not interfere with the cell system or with the specific cytokine-specific ELISA [40]. To carry out the interference test, a known contaminant must be used to perform the recovery rate. The spike recovery is characterized by exposing the test product to different concentrations of the contaminant, specifically a curve, and obtaining values between 50 and 200% of the control concentrations in the recovery of cytokine release. Despite the interference tests being part of the pyrogen test protocol in the Pharmacopoeia, from the selected studies, only Mazzotti et al. [22] carried out the standardized methodology to test the interferents in titanium clips, identifying no interference for these materials on the MAT.

Nevertheless, all authors used standard endotoxin stimuli, such as LPS from different strains, as positive controls. In addition, some authors investigated NEP stimuli, such as Zymosan [22,35,39], PGN [33,35], and LTA [4,35,37]. A review by Hartung et al. [46] discusses the detection of NEP as a fundamental advantage over the BET and the basis for the total replacement of the test in rabbits. This fact should be a guiding factor for selecting which MAT variant to use, as also discussed by Spreitzer et al. [30]. Hartung et al. [46] suggested creating a validation management group and designing the validation study to identify various test materials and non-endotoxin pyrogens. Brown et al. [15] pointed out the need for standardization of the positive control, despite the European Pharmacopoeia recommending the use of two non-endotoxin positive controls, and Petersen et al. [49] discuss characteristics to consider when selecting a positive control material for an in vitro assay.

#### 3.5.6. Correlations between the Rabbit Pyrogen Test (RPT), Limulus Amebocyte Lysate (LAL), and Monocyte Activation Test (MAT)

Although countries have already imposed the mandatory replacement of the pyrogen test in rabbits, the discussion on how to relate the results between the RPT, LAL, and MAT is still ongoing [15]. In this review, most of the studies (seven) compared the MAT with the LAL [4,22,32,33,34,35,36], four studies compared with the RPT [33,34,36,37], and only three studies performed the correlation of results between the three methods RPT, LAL, and MAT [33,34,36].

Haishima et al. [33] evaluated the relationship between pyrogenicity and bacterial endotoxin contamination in latex products, comparing the RPT< LAL and the MAT. The results showed that both gloves and catheters were pyrogenic in the RPT. In the MAT using MM6-CA8 cells, the samples were sensitive at doses greater than 10–100 pg/mL, inducing the production of IL-1β, IL-6, and TNF-α. The LAL assay showed intense activity for LPS at levels of 3.2 and 13.6 ng/mL, but it did not detect other bacterial and fungal cell wall components like Peptidoglycan and 1,3-β-D-glucans. Despite these differences, the authors concluded that all three methods effectively detected endotoxin-type pyrogens in biomaterials.

Nakagawa et al. [34] evaluated dressings of natural biomaterials in fresh human and rabbit blood matrices and MM8-CA8 cells compared to the LAL and RPT. In the LAL test, high concentrations of endotoxin were detected in extracts from three kinds of calcium alginate products, which also evoked fever in rabbits and induced the release of IL-6 from MM6-CA8.

Mohanan et al. [36] compared the LAL, RPT, and MAT evaluating gelatinous polymer materials. For the RPT, all evaluated biomaterials were pyrogenic according to the American Pharmacopoeia (USP) and ISO 10993-11:2017 methodologies [23]. The LAL indicated that the endotoxin levels were above the accepted threshold, while all samples of polymeric gelatin biomaterials induced IL-1β levels at a concentration of 4000 µg/mL above the threshold of 0.5 EU/mL, indicating pyrogenicity by the MAT threshold [36].

#### 3.5.7. Summary of Evidence

The present review identified studies analyzing the applicability of MAT for several medical biomaterials, including titanium, polymeric materials, bioglasses, swabs, and other ceramic materials, with a predominance of implantable materials. To test these materials, one of the biggest challenges concerning traditional MAT protocols arises from their solid nature, requiring adaptations in exposure to blood matrices. This includes the development of specific devices for sample immersion, variations in experimental conditions, such as time and blood volume, and methods for determining interferents. To perform MAT, studies used the most diverse cell source described for the MAT with a predominance of fresh blood. Results indicate that cryopreserved blood was more effective for metallic implant materials of titanium alloys.

The main cytokine evaluated was IL-1β, reflecting the main system already described in the pharmacopeias for the MAT. Still, other cytokines (IL-6 and TNF-alpha) were also evaluated, which greatly impacted the expected tissue regeneration for most of the investigated devices, bringing additional functionality to the method. The determination of interferents is another challenge regarding the evaluation of medical devices, especially considering that many may present intrinsic pyrogenicity to the material. The authors dealt, in general, with this determination from the spike recovery test, where different protocols were compared, including the immersion of the samples in LPS solutions and the addition of pyrogen solutions in direct contact with the surface of the material, where polymeric materials prove to be more efficient for the determination of LPS contamination. In the correlation of results between the RPT, LAL, and MAT, the authors concluded that the three methods can detect the presence of endotoxin-type pyrogens in biomaterials.

With this review, it became clear that many products still need to be tested to cover the broad scope of biomaterials and medical devices. On the other hand, essential generalizations and insights can be raised by the literature already available on the subject, such as the need to adapt the MAT test to different sizes and shapes of medical devices, as well as the investigation of the advantages of direct exposure of the test material by immersion or through eluate, as recommended for in vitro cytotoxicity tests in standards such as ISO 10993-12:2021 [50]. It would be necessary for international standards for the evaluation of biomaterials to invest in and promote the MAT and its applications, as the European Pharmacopoeia was concerned with the validation of the test for injectable products [46] to reach a new level of scientific advancement, and technology in the development of new medical materials, as well as in their safety assessments and regulatory release regarding their pyrogenic potential.

#### 3.5.8. Review Limitations

As a limitation of the present review, we acknowledge that the search strategy was not designed to comprehensively cover all databases, particularly regarding incomplete sources of evidence such as gray literature or those discussing the topic without providing primary experimental data. The international regulatory framework for the application of MAT for biomaterials and medical devices is still evolving, and the lack of standardized rules likely limits the scope of studies on the subject. Additionally, there is significant heterogeneity in methodologies and results. Biomaterials and medical devices encompass products with diverse chemical natures, making it challenging to apply conclusions and findings from one category of material to others.

## 4. Conclusions

This review has highlighted the significant progress made in applying the MAT for the detection of pyrogens in medical devices and biomaterials. The findings indicate that the MAT is suitable for a wide range of materials, including polymeric, metallic, and natural biomaterials. However, certain challenges remain, particularly regarding the adaptation of the MAT for specific material properties and testing conditions. The need for optimized protocols, including the use of direct contact and eluate methods, as well as dynamic incubation, was emphasized in several studies. Additionally, the choice of biological matrices, such as fresh or cryopreserved blood and monocytic cell lines, plays a crucial role in the sensitivity and reproducibility of the test results. Furthermore, some classes of materials, such as ceramics and calcium phosphates, are lacking evidence and validation for MAT in the literature, demanding further research. In conclusion, while MAT has shown great promise as a reliable method for pyrogen testing in medical devices and biomaterials, continued research and development are essential to address existing gaps and optimize the methodology for broader applications. Ensuring the standardization and validation of MAT will facilitate its adoption and improve the safety and efficacy of innovative medical technologies.

## Figures and Tables

**Table 1:** Search strategy.

Database	Search Strategy
PubMed	(pyrogen test OR “monocyte activation test”) AND (medical device* OR biomaterial*)
Scopus	TITLE-ABS-KEY ((pyrogen AND test OR “monocyte activation test”) AND (medical AND device* OR biomaterial*))
Web of Science	TS = (pyrogen) OR TS = (test) OR TS = (“monocyte activation test”) AND TS = (medical AND device*) OR TS = (biomaterial*)

**Table 2:** Main characteristics of the selected studies.

Author/Year	Material	Controls	Protocol of Exposure	Test Matrix	Pro-Inflammatory Cytokine	Comparisons with Other Tests
Haishima et al., 2001 [33]	Surgical gloves and catheters	LPS *E. coli* O55:B5; (JPSE) *E. coli* UKT-B; PGN *S. aureus*	Extract ^1^	MM6 cells	IL-1 β, IL-6, and TNF-α	LAL kinetic-chromogenic and RPT
Nakagawa et al., 2003 [34]	Natural biomaterial dressing (calcium alginate, collagen, quinine, and poly-L-leucine)	LPS *E. coli* 055:B5	Extract	Fresh blood (human and rabbit); MM6-CA8 cells	TNF-α and IL-6	LAL, RPT
Hasiwa et al., 2007 [35]	Steel and titanium implants; polystyrene and metal plates	LPS *E. coli* O113; *E. coli* O111:B4; LTA *S. aureus* (SaLTA); *B. subtilis* (BsLTA); Peptidoglycan (PGN): *S. aureus* (SaPGN); *B. subtilis* (BsPGN); *E. coli* (EcPGN) and Zymosan ^1^	Direct contact and extract	Fresh and cryopreserved blood	IL-1β	LAL chromogenic endpoint
Mazzotti et al., 2007 [22]	Titanium aneurysm clip	LPS *E. coli* O-113 and Zymosan A	Direct contact	Fresh and cryopreserved blood	IL-1β	LAL
Banerjee and Mohanan, 2011 [37]	Uninformed polymeric biomaterials	LPS from *E. coli* 055:B5; LTA from *B. subtilis*; 2,4,6-trinitrophenol, and PHA	Extract	Fresh blood	IL-1β	RPT
Mohanan et al., 2011 [36]	Gelatin polymeric materials	Endotoxin ^1^	Extract	Fresh blood	IL-1β	LAL and RPT
Harder et al., 2012 [38]	Titanium and zirconia dental implants	LPS ^1^	Direct contact	Fresh blood	(TLR4; TLR9; IL-1β; NF-kB; TNF- α; FADD); IL-1β	RT-qPCR
Stang et al., 2014 [4]	Steel plates, cobalt-chromium stents, and ePTFE vascular grafts	LPS *E. coli* O113:H10:K e LTA *S. aureus*	Direct contact and extract	Fresh blood	IL-1β	LAL, MAT, and modified MAT
Trunk et al., 2019 [39]	Cotton-based medical devices (swab)	LPS *E. coli* O113:H10:K; and Zymosan *S. cerevisiae*	Direct contact	Fresh blood or PBMC	Il-1β, IL-6	-
Werner et al., 2009 [32]	Intraocular lenses	*E. coli* O-111; *P. putida*; *S. epidermidis*	Extract	Fresh blood	IL-1β	LAL

^1^ not informed.

**Table 3:** Quality assessment of the selected studies according to the Toxicological Data Reliability Assessment Tool (ToxRTool) criteria.

Reference	Group I: Test Substance Identification	Group II: Test System	Group III: Study Design	Group IV: Study Results	Group V: Plausibility of Design and Data	Total	Reliability Categorization
Haishima et al., 2001 [33]	1	3	6	2	2	14	reliable with restriction
Nakagawa et al., 2003 [34]	2	3	6	3	2	16	reliable without restrictions
Hasiwa et al., 2007 [35]	2	3	5	3	1	14	reliable with restriction
Mazzotti, F. et al., 2007 [22]	3	3	5	2	2	15	reliable without restrictions
Banerjee and Mohanan, 2011 [37]	0	3	6	2	2	13	reliable with restriction
Mohanan et al., 2011 [36]	0	2	6	2	2	12	reliable with restriction
Harder et al., 2012 [38]	2	2	5	2	2	13	reliable with restriction
Stang et al., 2014 [4]	2	3	6	2	2	15	reliable without restrictions
Trunk et al., 2019 [39]	2	3	6	3	2	16	reliable without restrictions
Werner et al., 2009 [32]	2	3	6	3	1	15	reliable without restrictions

## Data Availability

The original contributions presented in this study are included in the article/Supplementary Materials; further inquiries can be directed to the corresponding author/s.

## Supplementary Materials

The following supporting information can be downloaded at: https://www.mdpi.com/article/10.3390/ijms25147844/s1. Reference [51] are cited in the Supplementary Materials.
